# Characteristics and Prognosis of Patients with Advanced Hepatocellular Carcinoma Treated with Atezolizumab/Bevacizumab Combination Therapy Who Achieved Complete Response

**DOI:** 10.3390/curroncol31100463

**Published:** 2024-10-16

**Authors:** Teiji Kuzuya, Naoto Kawabe, Hisanori Muto, Yoshihiko Tachi, Takeshi Ukai, Yuryo Wada, Gakushi Komura, Takuji Nakano, Hiroyuki Tanaka, Kazunori Nakaoka, Eizaburo Ohno, Kohei Funasaka, Mitsuo Nagasaka, Ryoji Miyahara, Yoshiki Hirooka

**Affiliations:** 1 Department of Gastroenterology and Hepatology, Fujita Health University, Toyoake 470-1192, Japan; kawabe@fujita-hu.ac.jp (N.K.); hisanori.muto@fujita-hu.ac.jp (H.M.); ytachi@fujita-hu.ac.jp (Y.T.); by-otonone@outlook.jp (T.U.); warbleman@gmail.com (Y.W.); wheeze0128@gmail.com (G.K.); tkjnkn@fujita-hu.ac.jp (T.N.); hiroyuki.tanaka@fujita-hu.ac.jp (H.T.); knakaoka@fujita-hu.ac.jp (K.N.); eizaburo.ono@fujita-hu.ac.jp (E.O.); k-funa@fujita-hu.ac.jp (K.F.); nmitsu@fujita-hu.ac.jp (M.N.); ryoji.miyahara@fujita-hu.ac.jp (R.M.); yoshiki.hirooka@fujita-hu.ac.jp (Y.H.); 2 Department of Gastroenterology and Hepatology, Fujita Health University Bantane Hospital, Nagoya 454-8509, Japan; 3 Department of Gastroenterology, Fujita Health University Okazaki Medical Center, Okazaki 444-0827, Japan

**Keywords:** complete response, hepatocellular carcinoma, atezolizumab, bevacizumab, transarterial chemoembolization, cancer-free, drug-off

## Abstract

Aim: To investigate the characteristics and prognosis of patients with advanced hepatocellular carcinoma (HCC) treated with atezolizumab and bevacizumab (Atz/Bev) who achieved a complete response (CR) according to the modified Response Evaluation Criteria in Solid Tumors (mRECIST). Methods: A total of 120 patients with Eastern Cooperative Oncology Group performance status (PS) 0 or 1 and Child–Pugh A at the start of Atz/Bev treatment were included. Barcelona Clinic Liver Cancer stage C was recorded in 59 patients. Results: The CR rate with Atz/Bev alone was 15.0%. The median time to CR was 3.4 months, and the median duration of CR was 15.6 months. A significant factor associated with achieving CR with Atz/Bev alone was an AFP ratio of 0.34 or less at 3 weeks. Adding transarterial chemoembolization (TACE) in the six patients who achieved a partial response increased the overall CR rate to 20%. Among the 24 patients who achieved CR, the median progression-free survival was 19.3 months, the median overall survival was not reached, and 14 patients (58.3%) were able to discontinue Atz/Bev and achieve a drug-free status. Twelve of these patients developed progressive disease (PD), but eleven successfully received post-PD treatments and responded well. Conclusions: Achieving CR by mRECIST using Atz/Bev alone or with additional TACE can be expected to offer an extremely favorable prognosis.

## 1. Introduction

In the IMbrave150 study [1], the combination of atezolizumab (an antibody against programmed cell death ligand) and bevacizumab (an antibody against vascular endothelial growth factor A) (Atz/Bev) in patients with advanced hepatocellular carcinoma (HCC) significantly prolonged overall survival (OS) and progression-free survival (PFS) compared with sorafenib [2], the standard of care. The complete response rate (CRR) and objective response rate (ORR) were also significantly higher compared with sorafenib. As a result, since 2020, Atz/Bev has been recommended as a first-line treatment option in the systemic therapy algorithm for advanced HCC [3,4,5].

In clinical practice, due to the high response rate to Atz/Bev, a treatment strategy is being considered in which local treatment is added as a conversion therapy and, once a “cancer-free status” is achieved, Atz/Bev is discontinued and the patient achieves a “drug-free status” [6,7,8]. Although many reports have examined Atz/Bev in clinical practice [8,9,10,11,12], there are no reports detailing the characteristics and prognosis of patients who achieve a complete response (CR).

The purpose of this study was to investigate the clinical background and outcomes in patients who achieved CR based on the modified Response Criteria in Solid Tumor Patients (mRECIST) [13] with or without additional transarterial chemoembolization (TACE).

## 2. Materials and Methods

### 2.1. Patients

From October 2020 to March 2024, Atz/Bev was introduced in 137 patients with advanced HCC who were ineligible for hepatectomy, radiofrequency ablation (RFA), or TACE at our institution. Of these, 17 patients with Child–Pugh B or Eastern Cooperative Oncology Group performance status (PS) 2 at the start of Atz/Bev treatment were excluded from the study. In total, 120 patients with Child–Pugh A and PS 0 or 1 at baseline were included in the study for the retrospective evaluation of outcomes.

### 2.2. Atz/Bev Treatment and Evaluation of Adverse Events

All patients received 1200 mg of intravenous Atz and 15 mg/kg body weight of Bev every 3 weeks. Adverse events (AEs) were evaluated according to the Common Terminology Criteria for Adverse Events (CTCAE), version 5.0 [14]. In the event of a drug-related AE, both or only one of Atz or Bev were temporarily discontinued until the AE resolved to grade 1 or 2 according to the manufacturer’s guidelines. Atz/Bev was continued until a potentially life-threatening AE occurred or the tumor progressed clinically.

### 2.3. Determination of the Antitumor Response

The antitumor response was assessed using the Response Evaluation Criteria in Solid Tumors (RECIST) version 1.1 [15] and modified RECIST (mRECIST). Dynamic computed tomography (CT) was performed at baseline, 6 weeks after initiation of Atz/Bev, and every 4–12 weeks thereafter, according to a predefined schedule. Since CR by RECIST is achieved in very few cases, we defined CR by mRECIST as “cancer-free status” in this study. “Drug-free status” was defined as being without Atz/Bev due to the achievement of CR by mRECIST for at least 12 weeks.

### 2.4. Evaluation of Changes in Tumor Markers

Serum alpha-fetoprotein (AFP), des-γ-carboxy-prothrombin (DCP), and lens culinaris agglutinin-reactive fraction of alpha-fetoprotein (AFP-L3) were measured as HCC tumor markers at the start of Atz/Bev treatment and at 3 and 6 weeks. Baseline concentrations were set to 1 for each patient, and the ratios of concentrations at 3 and 6 weeks from baseline were calculated. The analysis included patients with AFP ≥ 10 ng/mL, DCP ≥ 40 mAU/mL, and AFP-L3 ≥ 0.5%, excluding those with normal baseline levels.

### 2.5. Statistical Analysis

EZR version 1.29 (Saitama Medical University General Medical Center, Saitama, Japan) was used for the statistical analysis [16]. The Mann–Whitney *U* test compared the CR and non-CR groups for tumor marker ratios. Cutoff values were determined using the receiver operating characteristic (ROC) curve and area under the curve (AUC). A logistic regression analyzed the factors that were associated with CR. OS was defined as the time from Atz/Bev initiation to death or the last visit. PFS was defined as the time from Atz/Bev initiation to progression by RECIST, death, or the last visit. Time to CR by mRECIST was defined as the time from Atz/Bev initiation to CR identification. Duration of CR by mRECIST was defined as the time from CR identification to confirmed progression by RECIST. These durations were calculated using the Kaplan–Meier method and evaluated using the log-rank test. A *p*-value less than 0.05 was considered statistically significant.

## 3. Results

### 3.1. Baseline Characteristics

Table 1 shows the baseline characteristics of the 120 patients with HCC enrolled in the study who had PS 0 or 1 and Child–Pugh A at the start of Atz/Bev treatment. The median age of all subjects was 75 years, 100 were male, and 81 had a non-viral cause of HCC. PS score was 0 in 94 patients and 1 in 26 patients. Child–Pugh score was 5 in 85 patients and 6 in 35 patients. There were three cases of Barcelona Clinic Liver Cancer (BCLC) stage A, 58 cases of stage B, and 59 cases of stage C. The maximum tumor size (<30 mm, 30–50 mm, ≥50 mm) in BCLC stage A/B was 14, 24, and 23 cases, respectively, and 11, 29, and 19 cases in stage C (*p* = 0.5544). The number of tumors (1, 2–3, ≥4) was 3, 19, and 39 in stage A/B, respectively, and 1, 14, and 44 in stage C (*p* = 0.3631). The median AFP, DCP, and AFP-L3 values at baseline were 39.7 ng/mL (range: 1.8–233,543 ng/mL), 540 mAU/mL (range: 10–403,328 mAU/mL), and 15.6% (range: <0.5–99.6%), respectively. Atz/Bev was initiated as first-line systemic therapy in 83 patients. All patients started treatment with Atz/Bev at the recommended dose.

### 3.2. Outcomes with Atz/Bev Alone

Median follow-up was 14.5 months (range: 0.8–41.1 months). Median duration of Atz/Bev treatment was 7.6 months (95% confidence interval [CI]: 4.8–8.7 months).

Table 2 shows the best antitumor response by RECIST and mRECIST with Atz/Bev alone. According to RECIST, 1 patient (0.8%) achieved CR, 41 (34.2%) had a partial response (PR), 53 (44.2%) had stable disease (SD), 23 (19.2%) had progressive disease (PD), and 2 (1.7%) had not evaluated (NE), with CRR, ORR, and disease control rate (DCR) of 0.8%, 35.0%, and 79.2%, respectively. According to mRECIST, 18 patients (15.0%) achieved CR, 44 (36.7%) had PR, 33 (27.5%) had SD, 23 (19.2%) had PD, and 2 (1.7%) had NE, with a CRR, ORR, and DCR of 15.0%, 51.7%, and 79.2%, respectively.

**Figure 1:**
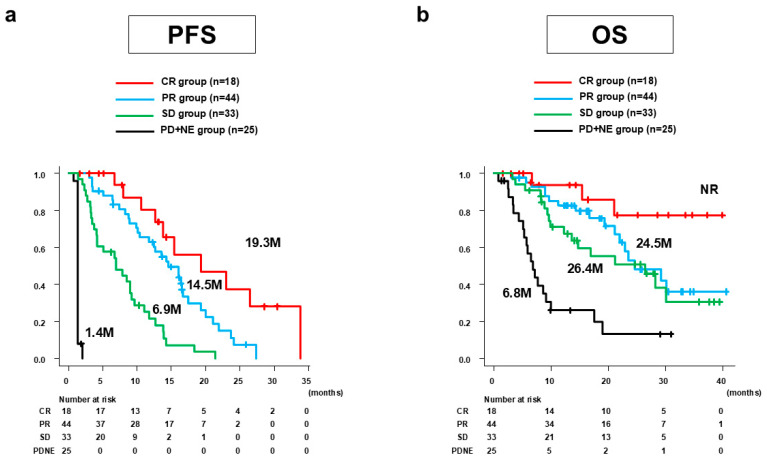
PFS and OS of Atz/Bev alone for all 120 patients stratified by the best antitumor response according to mRECIST. (**a**) Median PFS was 19.3 months (95%CI: 12.6 months–NR) for the CR group, 14.5 months (95%CI: 10.3–16.7) for the PR group, 6.9 months (95%CI: 4.1–9.2 months) for the SD group, and 1.4 months (95%CI: 1.4–1.4 months) for the PD + NE group. (**b**) Median OS was NR (95%CI: 21.0 months–NR) for the CR group, 24.5 months (95%CI: 21.1 months–NR) for the PR group, 26.4 months (95%CI: 12.2 months–NR) for the SD group, and 6.8 months (95%CI: 5.1–9.1 months) for the PD + NE group. PFS, progression-free survival; OS, overall survival; Atz/Bev, atezolizumab/bevacizumab; mRECIST, modified Response Evaluation Criteria in Solid Tumors; CI, confidence interval; NR, not reached; CR, complete response; PR, partial response; SD, stable disease; PD, progressive disease; NE, not evaluated; M, months.

Median PFS was 9.0 months (95%CI: 6.9–12.4 months). Figure 1a shows the PFS of Atz/Bev alone stratified by best antitumor response according to mRECIST. By group, the median PFS was 19.3 months (95%CI: 12.6 months–not reached [NR]) for the CR group, 14.5 months (95%CI: 10.3–16.7 months) for the PR group, 6.9 months (4.1–9.2 months) for the SD group, and 1.4 months (95%CI: 1.4–1.4 months) for the PD + NE group.

Median OS was 23.4 months (95%CI: 17.5–30.1 months). Figure 1b shows the OS of Atz/Bev alone stratified by the best antitumor response according to mRECIST. The median OS (95%CI) was NR (21.0 months–NR) for the CR group, 24.5 months (21.1 months–NR) for the PR group, 26.4 months (12.2 months–NR) for the SD group, and 6.8 months (5.1–9.1 months) for the PD + NE group.

### 3.3. Changes in Tumor Markers Early after Start of Atz/Bev in Patients Achieving CR by mRECIST with Atz/Bev Alone

The relationship between the tumor marker ratios at 3 and 6 weeks after Atz/Bev initiation and the best antitumor response of Atz/Bev alone according to mRECIST was examined in patients whose tumor markers were above the normal range at Atz/Bev initiation (Table 3). Patients who achieved CR by mRECIST (CR group) showed AFP ratios of 0.18 and 0.13 at Weeks 3 and 6, respectively, which were significantly lower than those of 0.87 and 0.92 in patients who did not achieve CR by mRECIST (non-CR group) (*p* = 0.0002 and *p* = 0.0003, respectively). Similarly, the DCP ratios for the CR group were 0.50 at 3 weeks and 0.32 at 6 weeks, which were significantly lower than 1.27 and 1.60 for the non-CR group (*p* = 0.0053 and *p* < 0.0001, respectively). On the other hand, the AFP-L3 ratios at Weeks 3 and 6 of Atz/Bev did not differ significantly between groups (*p* = 0.2366 and *p* = 0.0973, respectively).

ROC curves were plotted with CR achievement as the state variable and the AFP ratio, DCP ratio, and AFP-L3 ratio as the test variables. The results showed that the AFP ratio at 3 weeks, AFP ratio at 6 weeks, DCP ratio at 3 weeks, DCP ratio at 6 weeks, AFP-L3 ratio at 3 weeks, and AFP-L3 ratio at 6 weeks had AUCs of 0.906, 0.893, 0.743, 0.872, 0.619, and 0.664 as cutoff values for predicting CR, respectively. The AFP ratio at 3 weeks, DCP ratio at 6 weeks, and AFP-L3 ratio at 6 weeks were found to be optimal at 0.34, 0.59, and 0.87, respectively.

The relationship between tumor marker ratios at 3 and 6 weeks and the best antitumor response of Atz/Bev alone according to mRECIST, including patients with normal baseline markers, is shown in Supplementary Table S1. In the CR group, the AFP ratios were 0.70 at 3 weeks and 0.64 at 6 weeks, which were significantly lower than 0.87 and 0.93 in the non-CR group (*p* = 0.0016, *p* = 0.0004). Similarly, the DCP ratios were 0.69 and 0.36 in the CR group, compared with 1.33 and 1.56 in the non-CR group (*p* = 0.0014, *p* < 0.0001). There was no significant difference in the AFP-L3 ratios between the groups (*p* = 0.3285, *p* = 0.0607).

### 3.4. Factors Related to CR by mRECIST with Atz/Bev Alone

Table 4 shows the results of the univariate and multivariate analyses of the factors associated with CR by mRECIST in Atz/Bev alone. The univariate analysis revealed that the pre-treatment factor associated with achieving CR was a tumor number of fewer than four. During treatment, the factors linked to CR included an AFP ratio of ≤0.34 at 3 weeks and a DCP ratio of ≤0.59 at 6 weeks. According to the multivariate analyses, the AFP ratio at 3 weeks (≤0.34) was a significant and an independent factor associated with CR (odds ratio [OR], 52.63; 95%CI, 2.37–1000; *p* = 0.0122).

### 3.5. Outcomes with Atz/Bev + Additional TACE

Of the 44 patients who achieved PR by mRECIST with Atz/Bev alone, 6 received additional TACE aimed at CR. Five of those six patients achieved CR by mRECIST after one TACE session and one patient achieved CR by mRECIST after a total of two TACE sessions. Additional TACE increased the percentage of CR by mRECIST from 15.0% to 20.0% (Table 5).

**Figure 2:**
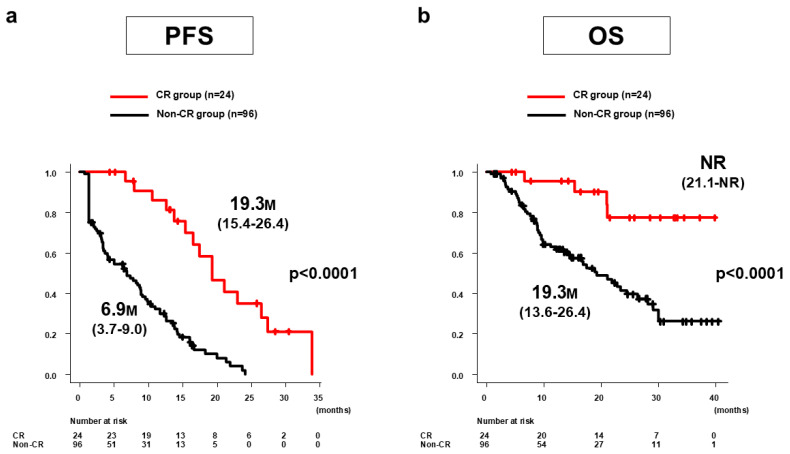
PFS and OS of Atz/Bev + additional TACE for all 120 patients in the CR and non-CR groups according to mRECIST. (**a**) Median PFS in the CR group (*n* = 24) was 19.3 months (95%CI: 15.4–26.4 months), significantly longer than the 6.9 months (95%CI: 3.7–9.0 months) in the non-CR group (*n* = 96) (*p* < 0.0001). (**b**) Median OS in the CR group was not reached (95%CI: 21.1 months–NR), significantly longer than the 19.3 months (95%CI: 13.6–26.4 months) in the non-CR group (*p* < 0.0001). PFS, progression-free survival; OS, overall survival; Atz/Bev, atezolizumab/bevacizumab; TACE, transarterial chemoembolization; CR, complete response; mRECIST, modified Response Evaluation Criteria in Solid Tumors; CI, confidence interval; NR, not reached; M, months.

The median PFS in the CR group (*n* = 24) was 19.3 months (95%CI: 15.4–26.4 months), which was significantly longer than the 6.9 months (95%CI: 3.7–9.0 months) in the non-CR group (*n* = 96, *p* < 0.0001) (Figure 2a). The median OS in the CR group was not reached (95%CI: 21.1 months–NR), which was significantly longer than the 19.3 months (95%CI: 13.6–26.4 months) in the non-CR group (*p* < 0.0001) (Figure 2b).

### 3.6. Outcomes of Patients Achieving CR by mRECIST

**Figure 3:**
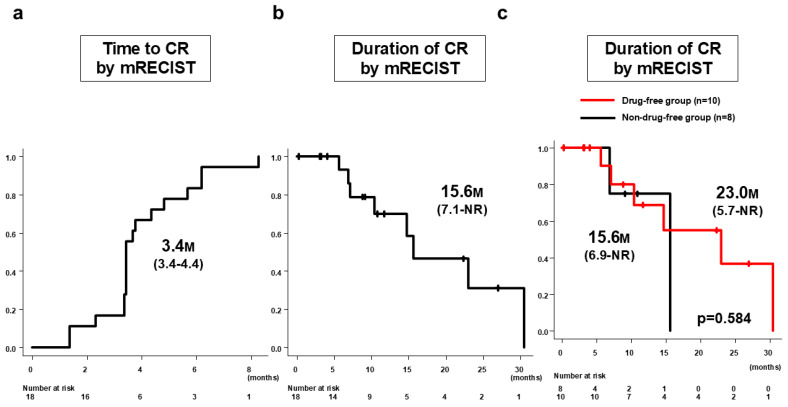
Time to CR by mRECIST and duration of CR by mRECIST in patients who achieved CR by Atz/Bev alone (*n* = 18). (**a**) Median time to CR by mRECIST was 3.4 months (95%CI: 3.4–4.4 months). (**b**) Duration of CR by mRECIST was 15.6 months (95%CI: 7.1 months–NR). (**c**) Median CR duration for the drug-free group (*n* = 10) was 23.0 months (95%CI: 5.7 months–NR months) and for the non-drug-free group (*n* = 8) was 15.6 months (95%CI: 6.9 months–NR), with no significant difference between the groups (*p* = 0.584). CR, complete response; mRECIST, modified Response Evaluation Criteria in Solid Tumors; Atz/Bev, atezolizumab/bevacizumab; CI, confidence interval.

**Figure 4:**
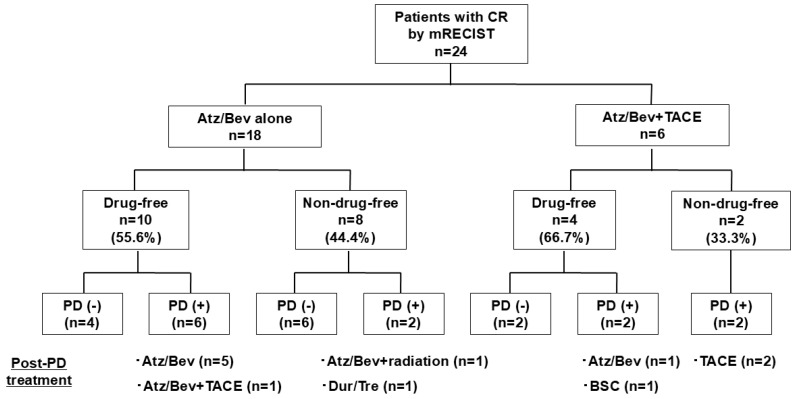
Clinical outcomes in patients who achieved CR by mRECIST—stratified by drug-free status. CR, complete response; mRECIST, modified Response Evaluation Criteria in Solid Tumors; Atz/Bev, atezolizumab/bevacizumab; PD, progressive disease; TACE, transarterial chemoembolization.

In the 18 patients who achieved CR by mRECIST with Atz/Bev alone, the median time to CR by mRECIST was 3.4 months (95%CI: 3.4–4.4 months) (Figure 3a). The duration of CR by mRECIST was 15.6 months (95%CI: 7.1 months–NR) (Figure 3b). Ten patients (drug-free group) discontinued Atz/Bev due to achieving CR by mRECIST and reached a drug-free status; the remaining eight patients (non-drug-free group) continued Atz/Bev after confirmation of CR. The median CR duration was 23.0 months (95%CI: 5.7 months–NR) for the drug-free group and 15.6 months (95%CI: 6.9 months–NR) for the non-drug-free group, showing no significant difference between the groups (*p* = 0.584) (Figure 3c). At cutoff, eight patients had PD; the post-PD treatment options included Atz/Bev alone in five patients, Atz/Bev + TACE in one patient, Atz/Bev + radiation in one patient, and durvalumab/tremelimumab in one patient (Figure 4).

In the 18 patients who achieved CR by mRECIST with Atz/Bev alone, at the time of the analysis, 8 patients had PD. Post-PD treatment options included Atz/Bev alone in five patients, Atz/Bev + TACE in one patient, Atz/Bev + radiation in one patient, and durvalumab/tremelimumab in one patient. Among the six patients who achieved CR by mRECIST with Atz/Bev + additional TACE, four patients showed PD. The post-PD treatment options included TACE in two patients, Atz/Bev alone in one patient, and best supporting care in one patient.

In the six patients who achieved CR by mRECIST with Atz/Bev + additional TACE, the duration of CR by mRECIST was 10.3 months (95%CI: 7.1 months–NR). Four patients discontinued Atz/Bev due to achieving CR by mRECIST and reached a drug-free status. The remaining two patients continued Atz/Bev after confirmation of CR. Median CR duration was 8.5 months (95%CI: 7.1 months–NR) for the drug-free group and 9.8 months (95%CI: 7.4 months–NR) for the non-drug-free group, showing no significant difference between groups (*p* = 0.586). At cutoff, four patients had PD; the post-PD treatment options included TACE in two patients, Atz/Bev alone in one patient, and best supporting care in one patient (Figure 4).

Of the 24 patients who achieved CR, 18 had all three tumor markers (AFP, DCP, AFP-L3) within the normal range. Of these, 12 patients maintained negative levels for all three markers for more than 24 weeks, but three of these patients relapsed. On the other hand, all six patients whose levels of one of the markers rose within 24 weeks relapsed, and three of the six patients whose levels of none of the markers became negative, also relapsed. Patients who remained negative for all three markers for 24 weeks or longer tended to have a longer median PFS (27.8 months) than the other patients (16.8 months) (*p* = 0.0863).

Table 6 shows the best antitumor response according to RECIST and mRECIST in the 12 patients treated with post-PD therapy. According to RECIST, CRR, ORR, and DCR were 0%, 16.7%, and 75.0%, respectively. According to mRECIST, CRR, ORR, and DCR were 16.7%, 33.3%, and 75.0%, respectively.

Grade 3 or higher AEs observed during the time to CR included proteinuria in five patients, hypertension in one patient, elevated AST/ALT levels in one patient, and duodenal perforation in one patient. Bevacizumab was temporarily discontinued in the five patients with proteinuria, and Atz/Bev treatment was permanently discontinued in the patient with duodenal perforation. There were no treatment interruptions or discontinuations in the remaining patients.

In the 24 patients who achieved CR by mRECIST, the median duration of Atz/Bev treatment was 12.6 months (95%CI: 7.6–17.0 months). The detailed reasons for the discontinuation of Atz/Bev treatment in the drug-free group (*n* = 14) were as follows: only achievement of CR by mRECIST in nine patients, AEs considered to be Atz/Bev-related in two patients (interstitial pneumonia in one patient, duodenal perforation in one patient), and other diseases in three patients (leukemia in one patient, cerebral hemorrhage in one patient, bacterial pneumonia in one patient). Of the ten patients in the non-drug-free group (*n* = 10), three were still on Atz/Bev treatment. Two were transferred to other hospitals and their status was unknown. The remaining five patients discontinued Atz/Bev for the following reasons: two patients had worsening hepatic function, one patient discontinued due to PD, one patient developed AEs considered to be Atz/Bev-related (one patient with colitis and Guillain–Barré syndrome), and one patient developed other diseases (aortic dissection).

## 4. Discussion

This is the first study to focus on the characteristics and outcomes of patients with advanced HCC who achieved CR after Atz/Bev treatment in a clinical setting. We found that 15% of patients achieved CR by mRECIST with Atz/Bev alone, with a median time to CR of 3.4 months and a median duration of CR of 15.6 months. A significant and independent factor associated with achieving CR with Atz/Bev alone was an AFP ratio of 0.34 or less at 3 weeks. In addition, six patients who achieved PR with Atz/Bev alone underwent TACE aimed at achieving CR, increasing the overall CRR to 20%. The prognosis for these patients was very good.

In the era of molecularly targeted agents (MTAs) alone, CRRs by RECIST were as low as <1.0% [2,17], but the IMbrave150 trial reported a CRR of 8% by RECIST and 12% by mRECIST in the Atz/Bev arm [1]. In the current study, the CRR by mRECIST with Atz/Bev alone was 15%, which was similar to the IMbrave150 study. In an exploratory analysis of the IMbrave150 trial [18], the median time to CR by RECIST was 7.0 months (range: 1.2–18.8 months) and the median time to CR by mRECIST was 5.5 months (range: 1.2–16.8 months). In the current study, the median time to CR by mRECIST was 3.4 months (95%CI: 3.4–4.4 months). The reason for the shorter time to CR of approximately 2 months in this study may be that in the IMbrave150 study, CR and PR were determined by two consecutive tumor assessments at least 28 days apart, whereas CR and PR were determined by a single tumor assessment in this study.

Baseline factors associated with a favorable antitumor response and prognosis have been reported [19,20,21]. In this study, having fewer than four tumors at baseline was associated with CR. However, this was a univariate analysis, and at this time it is difficult to identify useful factors that can predict CR cases prior to the initiation of Atz/Bev treatment. The identification of useful biomarkers that are predictive of an antitumor response prior to the initiation of therapy remains an important issue in treatment choice.

Several studies have explored the relationship between post-treatment tumor marker trends and antitumor response and prognosis with systemic therapy. Early AFP reduction with Atz/Bev treatment is reported to be associated with good outcomes [22,23,24,25], and this study found significant early AFP reduction in the CR group compared with the non-CR group. Unlike AFP, DCP trends during systemic therapy do not generally reflect antitumor efficacy due to the hypoxic state induced by VEGF inhibitors [22,26,27], which increases DCP production [28]. Thus, increased DCP can indicate either an effective antitumor response or tumor progression. In this study, an initial decrease in DCP was observed in CR cases, but only AFP reduction was a significant factor for CR in the multivariate analysis. Few studies have examined AFP-L3 trends during systemic therapy, and in this study, AFP-L3 ratios at Weeks 3 and 6 did not significantly differ between the CR and non-CR groups. An early and marked decrease in AFP may be a useful biomarker for predicting CR.

With the increase in the number of cases in which tumor shrinkage can be achieved with drug therapy, multidisciplinary treatment strategies are attracting attention in advanced HCC, aiming for cancer-free and drug-free statuses by adding local treatment to systemic therapy as the mainstay [6,7,8]. Kudo et al. conducted a multicenter, retrospective study to determine whether the addition of local therapy with curative intent is useful in increasing the CRR achieved in patients treated with Atz/Bev as the primary therapy for TACE-ineligible intermediate-stage HCC [8]. Of the 110 patients treated with Atz/Bev, 3 patients achieved CR with Atz/Bev alone and 35 patients were treated with radical therapy (hepatectomy in 7 patients, RFA in 13 patients, and TACE in 15 patients), resulting in CR (cancer-free status) in 38 patients (35%) overall. In this study, PR was achieved with mRECIST using Atz/Bev alone and TACE was performed in six patients with a reduced residual tumor volume, resulting in CR in all patients. RFA and TACE during immune checkpoint inhibitor (ICI) treatment are expected to enhance the antitumor effects of subsequent ICI treatment by activating the cancer immune cycle through the release of tumor-associated antigens, along with the tumor necrosis effect.

At the time when only MTAs were available, few cases of significant response were achieved. Even when CR was achieved, the risk of an undetectable residual tumor remained a concern, so most patients continued with the MTA as long as the AEs remained under control. The main concern with ICI therapy is the development of immune-related AEs (irAEs). Even after CR is achieved, the long-term use of ICIs carries a risk of serious irAEs. In the cases we studied, during the first 2 years of Atz/Bev use, we essentially continued Atz/Bev after achieving CR with mRECIST as long as the AEs remained within acceptable limits, as was our previous thinking. However, two of the patients who achieved CR with mRECIST and continued on Atz/Bev developed severe irAEs (one with colitis and Guillain–Barré syndrome, one with interstitial pneumonia). In light of this experience, we have recently changed our approach and started to suggest that our patients discontinue Atz/Bev in cases where CR was obtained on mRECIST, changing our previous stance. Kudo et al. proposed that the criteria for discontinuing the drug (drug-off criteria) should be: (1) mRECIST CR achieved by TACE or RFA; (2) Sustained normalization of all three tumor markers (AFP, DCP, AFP-L3) for 12–24 weeks or longer; (3) Contrast-enhanced echocardiography showing the loss of intra-tumoral blood flow [7]. In the aforementioned study by Kudo et al., 25 of the 110 patients (23%) were able to discontinue Atz/Bev because they had achieved CR at the time of the analysis. In our hospital, we generally follow the above criteria, but even if all the above criteria are not met, we discuss the risk of irAEs with the patient and discontinue Atz/Bev if the patient wishes. Fourteen patients (58.3%) who achieved CR discontinued Atz/Bev. In the cases we studied, very few met the drug-off criteria proposed by Kudo et al. The main reason for this was that these criteria had only recently been introduced and we were not fully aware of them. In addition, only a small number of cases achieved CR with additional local therapy (only six cases underwent TACE), and the treatment response was confirmed using contrast-enhanced ultrasound in only three cases. However, we were able to evaluate one of the criteria, namely whether the three tumor markers (AFP, DCP, ALP-L3) remained negative for 24 weeks or longer. Patients whose tumor markers remained negative for 24 weeks had fewer recurrences and better PFS. Because HCC is a multicentric disease, achieving a cure and preventing recurrence are challenging. However, our results suggest that maintaining tumor marker negativity for 24 weeks or longer may be a useful indicator when considering treatment discontinuation.

On the other hand, the main concern regarding the discontinuation of Atz/Bev therapy is the recurrence of HCC. In this study, eight (57.1%) of the fourteen patients who achieved CR and discontinued Atz/Bev developed PD due to occurrence of a new lesion, but seven of those patients could be retreated for recurrence and showed a good response to therapy. Bevacizumab is also an anti-VEGF inhibitor and long-term use increases the risk of developing severe proteinuria [9]. If severe proteinuria becomes irreversible, VEGF inhibitors may not be the treatment of choice for relapse [9,29]. Based on the results of this study, we believe that it is appropriate to consider a no-treatment strategy that essentially discontinues Atz/Bev after CR is achieved, rather than continuing Atz/Bev treatment in a haphazard way. However, this depends on the patient’s wishes and circumstances, and a study of a larger number of cases is needed to clarify this.

Several reports have described patients achieving a cancer-free status and demonstrating a favorable prognosis with conversion therapy during Atz/Bev treatment [8,30,31,32,33]. The most radical local treatment for conversion therapy is hepatectomy. However, hepatectomy is generally more invasive than RFA or TACE. Some patients, especially older patients, are hesitant to undergo hepatectomy even when it is recommended. In this regard, TACE is minimally invasive, and patients are more likely to undergo this procedure due to its lower threshold. Additionally, using TACE as needed during systemic therapy can be beneficial in several ways: (1) It allows for a detailed imaging evaluation of residual disease through the combination of CT arterial portography and CT hepatic arteriography prior to TACE; (2) It helps to reduce the tumor volume through embolization; (3) It promotes the antitumor effects of subsequent ICI therapy by releasing necrotic cancer antigens into the blood through embolization; (4) The accumulation of lipiodol in residual disease after TACE enables a more reliable CR assessment and facilitates an easier transition to a drug-free status.

In this study, the median PFS for the 18 patients who achieved CR with Atz/Bev alone was 19.3 months, and OS was not reached. Including six additional patients who achieved CR with TACE, the total of twenty-four patients showed similarly favorable outcomes, with a median PFS of 19.3 months and OS not reached. Achieving CR with systemic therapy alone and achieving CR with a combination of systemic therapy and local treatment have fundamentally different implications. However, from these results, achieving CR with either Atz/Bev alone or with TACE resulted in a highly favorable prognosis. While the efficacy of systemic therapy has improved, there are limitations when it is used alone. The integration of additional local treatments, such as TACE, to achieve CR as needed during systemic therapy may represent a valuable strategy to overcome the inherent limitations of systemic therapy alone.

This study had several limitations. First, it was a retrospective, non-randomized analysis. Approximately 50% of the patients were classified as BCLC stage C, which is lower than in most clinical trials and may have contributed to the favorable prognosis and antitumor response observed. Additionally, the small sample size and short follow-up period, particularly in assessing long-term outcomes, were notable limitations. There may have been selection bias in the ancillary treatments, such as TACE, and the timing of Atz/Bev discontinuation after CR based on the mRECIST criteria was not standardized. Moreover, the tumor marker analysis was limited to patients with elevated baseline levels, making it difficult to draw conclusions for those with normal levels. Larger, independent studies are needed to validate these findings.

## 5. Conclusions

In conclusion, CR by mRECIST with Atz/Bev alone was achieved in 15% of patients, with marked reductions in AFP at 3 weeks after the initiation of Atz/Bev therapy. Achievement of CR by mRECIST with Atz/Bev alone or with additional TACE can be expected to have an extremely favorable prognosis.

## Figures and Tables

**Table 1:** Baseline characteristics at atezolizumab/bevacizumab initiation.

Patient Characteristics	*n* = 120
Median age (years, range)	75 (38–90)
Sex (men/women)	100/20
Etiology (HBV/HCV/non-viral)	15/24/81
Prior treatments for HCC (−/+)	38/82
PS (0/1)	94/26
Child–Pugh score (5/6)	85/35
mALBI grade (1/2a/2b) (*n*)	47/36/37
BCLC stage (A/B/C) (*n*)	3/58/59
Tumor size (<30 mm/30–50 mm/≥50 mm)	25/53/42
Number of tumors (1/2–3/≥4)	4/33/83
Liver occupation rate 50% (<50%/≥50%)	103/17
Portal vein invasion (0/1/2/3/4)	99/2/7/5/7
Extrahepatic spread (−/+)	76/44
Median AFP level (ng/mL, range)	39.7 (1.8–233,543)
Median DCP level (mAU/mL, range)	540 (10–403,328)
Median AFP-L3 level (%, range)	15.6 (<0.5–99.6)
NLR (range)	2.47 (0.59–14.19)
Treatment line (1st/2nd/3rd/4th)	83/35/1/1

HBV, hepatitis B virus; HCV, hepatitis C virus; HCC, hepatocellular carcinoma; non-viral, non-hepatitis B or C; PS, performance status; mALBI, modified albumin–bilirubin; BCLC, Barcelona Clinic Liver Cancer; AFP, alpha-fetoprotein; DCP, des-γ-carboxy prothrombin; AFP-L3, lens culinaris agglutinin-reactive fraction of alpha-fetoprotein; NLR, neutrophil-to-lymphocyte ratio.

**Table 2:** Best antitumor response to atezolizumab/bevacizumab alone according to RECIST and mRECIST (*n* = 120).

	CR	PR	SD	PD	NE	CRR	ORR	DCR
	*n* (%)	*n* (%)	*n* (%)	*n* (%)	*n* (%)			
RECIST	1	41	53	23	2	0.8%	35.0%	79.2%
	(0.8)	(34.2)	(44.2)	(19.2)	(1.7)			
mRECIST	18	44	33	23	2	15.0%	51.7%	79.2%
	(15.0)	(36.7)	(27.5)	(19.2)	(1.7)			

RECIST, Response Evaluation Criteria in Solid Tumors; mRECIST, modified RECIST; CR, complete response; PR, partial response; SD, stable disease; PD, progressive disease; NE, not evaluated; CRR, complete response rate; ORR, objective response rate; DCR, disease control rate.

**Table 3:** Relationship between the tumor marker ratios at 3 and 6 weeks after Atz/Bev initiation and the best antitumor response of Atz/Bev alone according to mRECIST in patients whose tumor markers were above the normal range at Atz/Bev initiation.

Tumor Marker Ratio		CR	PR	SD	PD + NE	Non-CR	*p* Value
		Median	Median	Median	Median	Median	(CR vs. Non-CR)
		(SE)	(SE)	(SE)	(SE)	(SE)	
AFP ratio	At 3 weeks	0.18	0.65	0.94	1.53	0.87	0.0002
		(0.09)	(0.05)	(0.08)	(0.16)	(0.07)	
	At 6 weeks	0.13	0.65	0.92	2.33	0.92	0.0003
		(0.11)	(0.08)	(0.14)	(0.30)	(0.12)	
DCP ratio	At 3 weeks	0.50	1.04	1.27	2.07	1.27	0.0053
		(0.19)	(0.29)	(0.36)	(2.23)	(0.70)	
	At 6 weeks	0.32	1.41	1.67	2.47	1.60	<0.0001
		(0.14)	(1.53)	(0.57)	(2.31)	(0.95)	
AFP-L3 ratio	At 3 weeks	0.98	1.00	1.01	1.00	1.00	0.2366
		(0.06)	(0.01)	(0.06)	(0.09)	(0.03)	
	At 6 weeks	0.87	0.97	1.01	1.06	1.00	0.0973
		(0.14)	(0.07)	(0.10)	(0.09)	(0.04)	

Atz/Bev, atezolizumab/bevacizumab; mRECIST, modified Response Evaluation Criteria in Solid Tumors; CR, complete response; PR, partial response; SD, stable disease; PD, progressive disease; NE, not evaluated; SE, standard error; AFP, alpha fetoprotein; DCP, des-γ-carboxy prothrombin; AFP-L3, lens culinaris agglutinin-reactive fraction of alpha-fetoprotein.

**Table 4:** Univariate and multivariate analyses of factors associated with CR by mRECIST in Atz/Bev alone.

Factors	Univariate Analysis		Multivariate Analysis	
	OR (95%CI)	*p* Value	OR (95%CI)	*p* Value
Age (≥75 years)	1.92 (0.67–5.52)	0.2240		
Sex (men)	3.90 (0.49–31.08)	0.1999		
Etiology (non-viral)	0.54 (0.20–1.52)	0.2450		
Prior treatments for HCC (−)	1.09 (0.38–3.18)	0.8690		
PS (1)	1.04 (0.31–3.48)	0.9505		
Child–Pugh score (6)	0.65 (0.20–2.15)	0.4844		
mALBI grade (G2b or 3)	0.84 (0.28–2.56)	0.7610		
BCLC stage (C)	1.35 (0.49–3.70)	0.5574		
Tumor size (<50 mm)	0.48 (0.17–1.32)	0.1535		
Number of tumors (<4)	3.47 (1.24–0.97)	0.0177	3.31 (0.21–52.11)	0.3948
Liver occupation rate 50% (≥50%)	0.80 (0.20–3.10)	0.7419		
Portal vein invasion (−)	4.15 (0.52–33.04)	0.1792		
Extrahepatic spread (+)	1.47 (0.53–4.05)	0.4594		
AFP level (≥400 ng/mL)	0.84 (0.25–2.77)	0.7681		
DCP level (≥400 mAU/mL)	0.58 (0.20–1.65)	0.3061		
AFP-L3 level (<10%)	1.62 (0.59–4.42)	0.3504		
NLR (≥2.47)	0.50 (0.17–1.44)	0.1974		
Treatment line (2nd or later)	1.53 (0.54–4.32)	0.4243		
AFP ratio at 3 weeks (≤0.34)	62.50 (8.85–500)	<0.0001	52.63 (2.37–1000)	0.0122
DCP ratio at 6 weeks (≤0.59)	31.25 (6.14–166.67)	<0.0001	4.46 (0.20–100)	0.3470
AFP-L3 ratio at 6 weeks (≤0.87)	44.20 (0.99–17.86)	0.0515		

Atz/Bev, atezolizumab/bevacizumab; CR, complete response; mRECIST, modified Response Evaluation Criteria in Solid Tumors; OR, odds ratio; CI, confidence interval; non-viral, non-hepatitis B or C; PS, performance status; mALBI, modified albumin–bilirubin; BCLC, Barcelona Clinic Liver Cancer; AFP, alpha fetoprotein; DCP, des-γ-carboxy prothrombin; AFP-L3, lens culinaris agglutinin-reactive fraction of alpha-fetoprotein; NLR, neutrophil-to-lymphocyte ratio.

**Table 5:** Best antitumor response to Atz/Bev + additional TACE according to RECIST and mRECIST (*n* = 120).

	CR	PR	SD	PD	NE	CRR	ORR	DCR
	*n* (%)	*n* (%)	*n* (%)	*n* (%)	*n* (%)			
RECIST	1	44	50	23	2	0.8%	37.5%	79.2%
	(0.8)	(36.7)	(41.7)	(19.2)	(1.7)			
mRECIST	24	38	33	23	2	20.0%	51.7%	79.2%
	(20.0)	(31.7)	(27.5)	(19.2)	(1.7)			

Atz/Bev, atezolizumab/bevacizumab; TACE, transarterial chemoembolization; RECIST, Response Evaluation Criteria in Solid Tumors; mRECIST, modified RECIST; CR, complete response; PR, partial response; SD, stable disease; PD, progressive disease; NE, not evaluated; CRR, complete response rate; ORR, objective response rate; DCR, disease control rate.

**Table 6:** Best antitumor response to post-PD treatment according to RECIST and mRECIST (*n* = 12).

	CR	PR	SD	PD	NE	CRR	ORR	DCR
	*n* (%)	*n* (%)	*n* (%)	*n* (%)	*n* (%)			
RECIST	0	4	5	2	1	0%	16.7%	75.0%
	(0)	(33.3%)	(41.7%)	(16.7%)	(8.3%)			
mRECIST	2	2	5	2	1	16.7%	33.3%	75.0%
	(16.7%)	(16.7%)	(41.7%)	(16.7%)	(8.3%)			

PD, progressive disease; RECIST, Response Evaluation Criteria in Solid Tumors; mRECIST, modified RECIST; CR, complete response; PR, partial response; SD, stable disease; NE, not evaluated; CRR, complete response rate; ORR, objective response rate; DCR, disease control rate.

## Data Availability

All the data generated or analyzed in this study are included in this article. Please direct any inquiries to the corresponding author.

## Supplementary Materials

The following supporting information can be downloaded at: https://www.mdpi.com/article/10.3390/curroncol31100463/s1, Table S1: Relationship between tumor marker ratios at 3 and 6 weeks after Atz/Bev initiation and best antitumor response of Atz/Bev alone according to mRECIST, including patients with normal baseline markers.
