# Supplementary material for: Characteristics and Prognosis of Patients with Advanced Hepatocellular Carcinoma Treated with Atezolizumab/Bevacizumab Combination Therapy Who Achieved Complete Response
Source: Curr Oncol. 2024 Oct 16;31(10):6218–31. doi: 10.3390/curroncol31100463 (PMC11506828; doi:10.3390/curroncol31100463)
Supplement: Supplementary file 1 [file curroncol-31-00463-s001.zip › curroncol-3212550-supplementary.pdf]

Supplementary Table S1. Relationship between tumor marker ratios at 3 and 6 weeks after Atz/Bev initiation and best antitumor response of Atz/Bev alone according to mRECIST, including patients with normal baseline markers.

Tumor marker ratio		CR median (SE)	PR median (SE)	SD median (SE)	PD+NE median (SE)	Non-CR median (SE)	*p* value (CR vs Non-CR)
AFP ratio	At 3 weeks	0.70 (0.08)	0.79 (0.04)	0.93 (0.06)	1.26 (0.12)	0.87 (0.04)	0.0016
	At 6 weeks	0.64 (0.09)	0.81 (0.05)	0.98 (0.11)	1.32 (0.24)	0.93 (0.08)	0.0004
DCP ratio	At 3 weeks	0.69 (0.16)	1.04 (0.25)	1.50 (0.49)	2.07 (2.03)	1.33 (0.57)	0.0014
	At 6 weeks	0.36 (0.12)	1.41 (1.23)	1.67 (0.68)	2.43 (2.05)	1.56 (0.78)	<0.0001
AFP-L3 ratio	At 3 weeks	1.00 (0.05)	1.00 (0.05)	1.01 (0.98)	1.00 (1.37)	1.00 (0.46)	0.3285
	At 6 weeks	1.00 (0.13)	1.00 (0.05)	1.01 (3.65)	1.01 (1.63)	1.00 (1.18)	0.0607

Atz/Bev, atezolizumab/bevacizumab; mRECIST, modified Response Evaluation Criteria in Solid Tumors; CR, complete response; PR, partial response; SD, stable disease; PD, progressive disease; NE, not evaluated; SE, standard error; AFP, alpha fetoprotein; DCP, des-γ-carboxy prothrombin; AFP-L3, lens culinaris agglutinin-reactive fraction of alpha-fetoprotein.
